# Chlamydia trachomatis Subverts Alpha-Actinins To Stabilize Its Inclusion

**DOI:** 10.1128/spectrum.02614-22

**Published:** 2023-01-18

**Authors:** A. Haines, J. Wesolowski, F. Paumet

**Affiliations:** a Department of Immunology and Microbiology, Thomas Jefferson University, Philadelphia, Pennsylvania, USA; LSU Health New Orleans

**Keywords:** *Chlamydia trachomatis*, actin, actin-binding proteins, cytoskeleton, host-pathogen interactions

## Abstract

Chlamydia trachomatis is the leading cause of sexually transmitted bacterial disease and a global health burden. As an obligate intracellular pathogen, Chlamydia has evolved many strategies to manipulate its host and establish its intracellular niche called the inclusion. C. trachomatis reorganizes the host actin cytoskeleton to form scaffolds around the inclusion and reinforce the growing inclusion membrane. To control the kinetics and formation of actin scaffolds, Chlamydia expresses the effector InaC/CT813, which activates the host GTPase RhoA. Here, we have discovered that InaC stabilizes actin scaffolds through the host actin cross-linking proteins α-actinins 1 and 4. We demonstrate that α-actinins are recruited to the inclusion membrane in an InaC-dependent manner and associate with actin scaffolds that envelop the inclusion. Small interfering RNA (siRNA)-mediated knockdown of α-actinins differentially regulate the frequency of actin scaffolds and impair inclusion stability, leaving them susceptible to rupture and to nonionic detergent extraction. Overall, our data identify new host effectors that are subverted by InaC to stabilize actin scaffolds, highlighting the versatility of InaC as a key regulator of the host cytoskeletal network during Chlamydia infection.

**IMPORTANCE** Despite antibiotics, recurrent C. trachomatis infections cause significant damage to the genital tract in men and women. Without a preventative vaccine, it is paramount to understand the virulence mechanisms that Chlamydia employs to cause disease. In this context, manipulation of the host cytoskeleton is a critical component of Chlamydia development. Actin scaffolds reinforce the integrity of Chlamydia’s infectious vacuole, which is a critical barrier between Chlamydia and the host environment. Having previously established that InaC co-opts RhoA to promote the formation of actin scaffolds around the inclusion, we now show that Chlamydia hijacks a new class of host effectors, α-actinins, to cross-link these scaffolds and further stabilize the inclusion. We also establish that a core function of the chlamydial effector InaC is the regulation of cytoskeletal stability during Chlamydia infection. Ultimately, this work expands our understanding of how bacterial pathogens subvert the actin cytoskeleton by targeting fundamental host effector proteins.

## INTRODUCTION

Chlamydia trachomatis is an obligate intracellular human-restricted pathogen that is the most common cause of bacterially sexually transmitted infections worldwide (1). C. trachomatis infection can lead to chronic inflammatory disease and ectopic pregnancy and is the leading cause of infectious blindness, called trachoma (2, 3). A unique feature of Chlamydia development is its biphasic life cycle (4). Infectious, but transcriptionally inactive elementary bodies (EBs) attach to host cells and are internalized into a membrane-bound vacuole, called the inclusion. Once internalized, EBs differentiate into noninfectious, transcriptionally active reticulate bodies that divide and synthesize bacterial effectors. As the inclusion develops, chlamydial effectors are secreted into the host microenvironment as well as onto the surface of the inclusion membrane. Here, these bacterial effectors appropriate the host cell to protect the inclusion, which is a key event in regulating C. trachomatis pathogenicity and survival (4).

Throughout its life cycle, Chlamydia hijacks the host cytoskeleton to promote its survival. Of particular importance is the formation of the functionally and kinetically distinct microtubule (MT) and actin scaffolds that surround the inclusion. Around 12 h postinfection (hpi), Chlamydia generates MT scaffolds (5), which are posttranslationally modified (PTM) at ~18 to 24 hpi (6). PTM-MT scaffolds are critical for the recruitment of the host Golgi apparatus, an important event for nutrient acquisition by C. trachomatis (6, 7). At ~32 to 40 hpi, actin scaffolds are woven around the inclusion to maintain its integrity as it expands (8). We and others have shown that the chlamydial effector InaC/CT813 is required for forming PTM-MT and actin scaffolds during C. trachomatis infection (9–11). Blocking actin scaffold formation by knocking out InaC or using actin-depolymerizing agents results in the premature lysis of inclusions (8, 11), highlighting their role in Chlamydia development. Recently, we found that InaC controls the formation of actin scaffolds by activating the small host GTPase RhoA (8, 11). As the primary function of InaC is to stabilize MT scaffolds, we assessed whether InaC is also involved in the stabilization of actin scaffolds. To do so, we focused our attention on a critical family of actin-binding proteins, the α-actinins.

α-Actinins are members of a superfamily of actin-binding proteins, which stabilize F-actin via cross-linking (12, 13). α-Actinins are comprised of four isoforms that share high nucleotide and amino acid sequence homology (14). They form antiparallel homodimers, which is driven by interactions between their spectrin-like repeat rod domains. α-Actinin homodimers coalesce to form dumbbell-shaped actin-binding domains at the N terminus of each monomer, which drives cross-linking of actin filaments (14, 15). They contribute to diverse cellular processes like cytokinesis, cell adhesion, and motility and, more recently, the regulation of transcriptional activity (13). While α-actinins are commonly associated with the hijacking of actin by bacterial pathogens (16, 17), their role during these infections is poorly understood.

Here, we identify α-actinins 1 and 4 as important regulators of actin scaffold stability during C. trachomatis infection. α-Actinins 1 and 4 are recruited to the inclusion in an InaC-dependent manner late during infection. While the depletion of α-actinins 1 and 4 differentially affect the prevalence of actin scaffolds, they are both required to stabilize these cytoskeletal scaffolds and consequently the inclusion. This requirement is evidenced by premature inclusion lysis and increased susceptibility to detergent extraction in the absence of α-actinins 1 and 4. Together, these data indicate that Chlamydia hijacks host α-actinins to stabilize actin scaffolds and reinforce the inclusion membrane. Ultimately, this work expands our understanding of InaC as a molecular platform for the manipulation of the host cytoskeleton during Chlamydia infection.

## RESULTS

### α-Actinins are recruited to the inclusion in an InaC-dependent manner late during infection.

α-Actinins are actin-binding proteins that promote actin bundling and stability (12–14). While α-actinins 1 and 4 are ubiquitously expressed, α-actinins 2 and 3 are muscle specific (14). Therefore, we excluded α-actinins 2 and 3 from this study and focused our attention on α-actinins 1 and 4.

**FIG 1:**
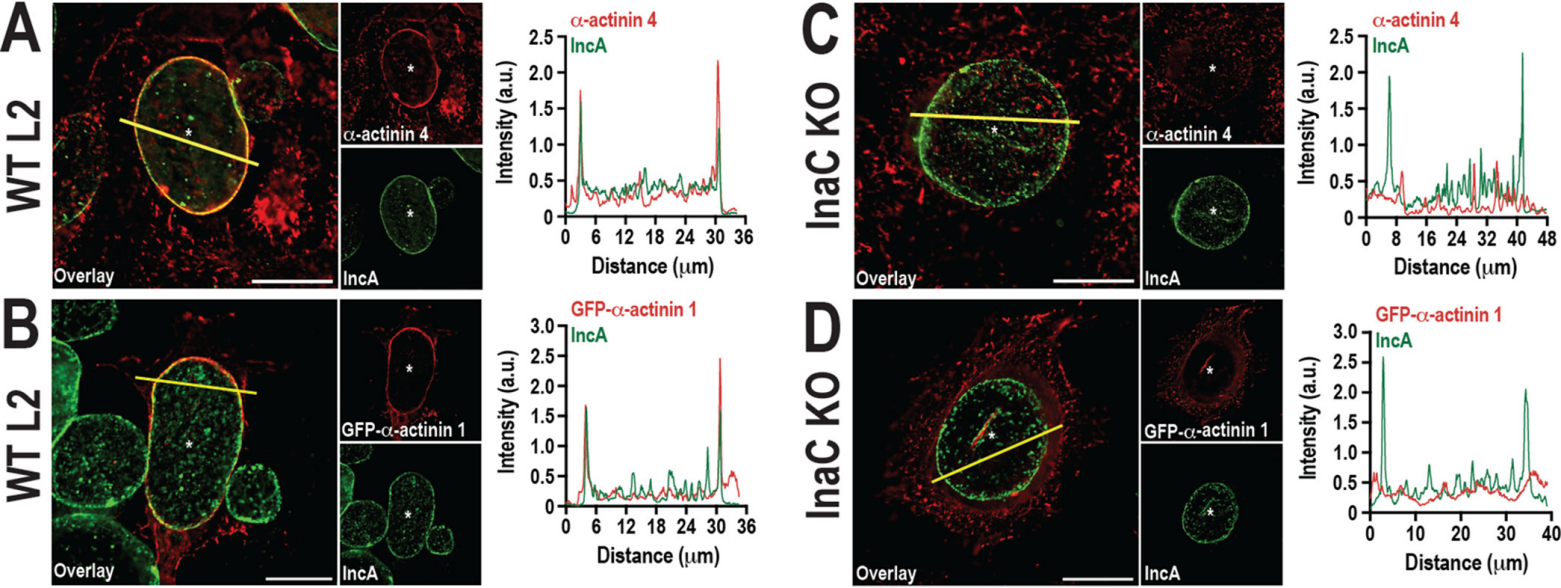
α-Actinins 1 and 4 are recruited to the inclusion in an InaC-dependent manner. Cells were infected with WT (A, B) or InaC KO (C, D) C. trachomatis L2 at an MOI of 1. (A, C) Infected cells were fixed and labeled with anti-α-actinin 4 (red) and anti-IncA (green) antibodies at 46 hpi. (B, D) For α-actinin 1 localization, cells were transfected with pEGFP-α-actinin 1 DNA at 24 hpi. Cells were then fixed at 46 hpi and labeled with anti-IncA (green) antibody. Asterisks denote inclusions. Scale bar, 20 μm. The line intensity scans display colocalization of α-actinin 4 or GFP-α-actinin 1 with IncA, which is a marker of the inclusion membrane. α-Actinins 4 and 1 are shown in red, and IncA is shown in green. The yellow line represents the path of the line scan through the inclusion.

First, we determined whether α-actinins 1 and 4 were recruited to the inclusion membrane using immunofluorescence microscopy. Cells were infected with wild-type (WT) C. trachomatis L2 and fixed at different times postinfection. α-Actinin 1 recruitment was assessed by transfecting cells with pEGFP-α-actinin 1, while endogenous α-actinin 4 recruitment was assessed using antibody. The cells were also labeled with anti-IncA antibody to delineate the inclusion membrane. We can detect both α-actinins around the inclusion at 46 h postinfection (hpi) (Fig. 1A and B). Next, we determined the kinetics of recruitment of α-actinin 4 on the inclusion. At 32 hpi, ~8% of the inclusions are α-actinin 4 positive (see Fig. S1A and C in the supplemental material). α-Actinin 4 recruitment further increases and plateaus between 40 and 48 hpi, where ~22% and ~25% of the inclusions are α-actinin 4 positive, respectively. The degree and timing of α-actinin 4 recruitment to the inclusion mirror those of actin scaffold formation, which also occurs late during infection (10, 11). In fact, we observed routinely that ~85% of inclusions with actin scaffolds colabeled with α-actinin 4 (data not shown). We could determine only the kinetics and distribution of recruitment for α-actinin 4, as we were unable to label endogenous α-actinin 1. However, considering the similarity between isoforms, α-actinin 1 likely follows a similar pattern.

Actin scaffold formation requires the chlamydial inclusion membrane protein InaC (9, 11). To test whether InaC is required for α-actinin recruitment to the inclusion, we infected cells with InaC knockout (KO) C. trachomatis L2 as described in Fig. 1 and analyzed α-actinin recruitment by immunofluorescence microscopy. In the absence of InaC, α-actinin 1 and 4 recruitment to the inclusion was impaired (Fig. 1C and D; Fig. S1B and C). α-Actinin 4 recruitment is rescued by complementing the InaC KO with InaC-FLAG on a plasmid (11) (see Fig. S2 in the supplemental material). As expected, complementation with InaC-FLAG restored the frequency of actin:α-actinin 4 double-positive inclusions to WT L2 levels (data not shown). Overall, these data demonstrate that the recruitment of α-actinins to the inclusion requires the chlamydial effector InaC.

### α-Actinin 4 is recruited on actin scaffolds and the inclusion membrane.

**FIG 2:**
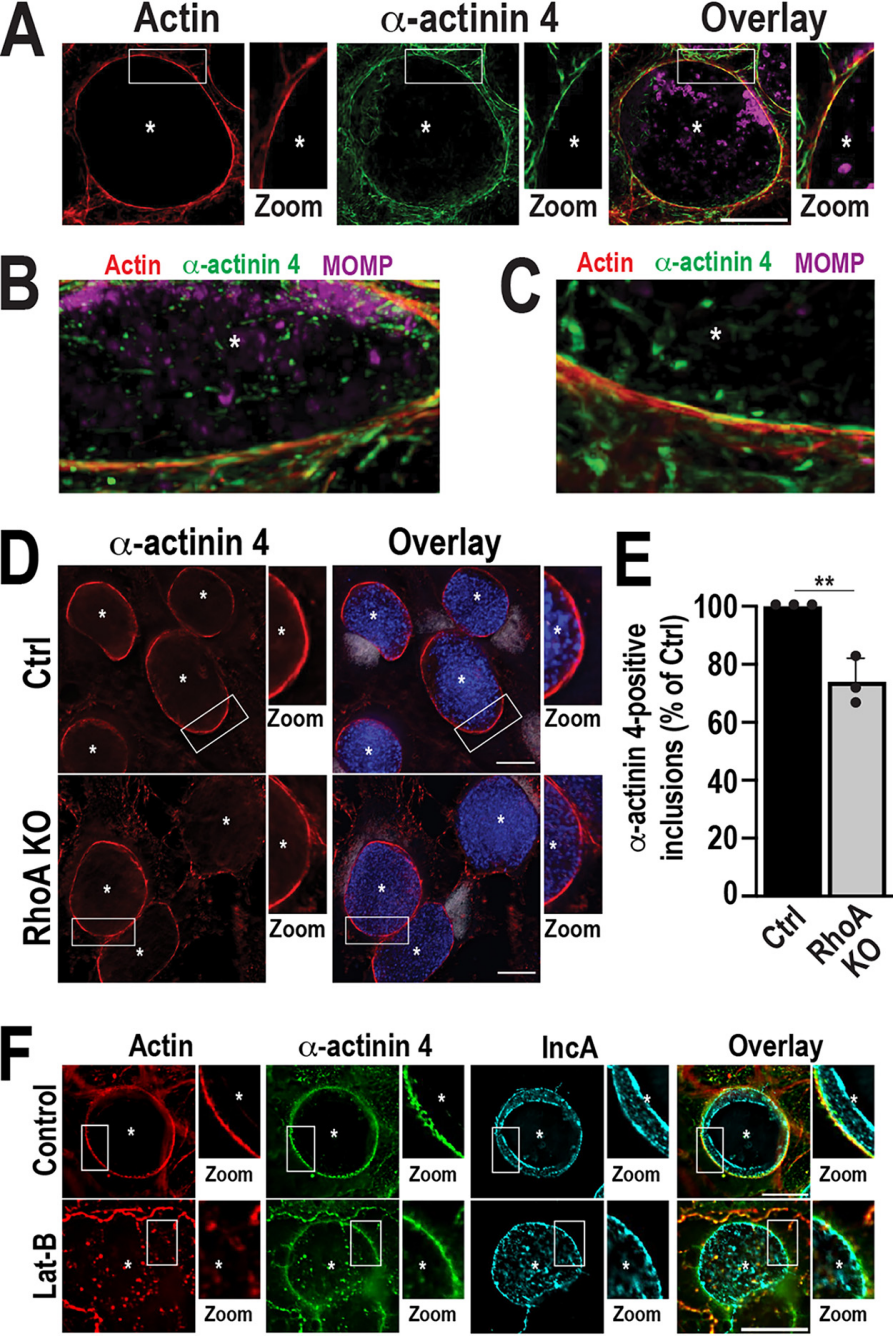
α-Actinin 4 is recruited on actin scaffolds and the inclusion membrane. (A to C) Cells were infected with WT C. trachomatis L2 at an MOI of 2. Cells were fixed at 56 hpi and labeled with phalloidin (red), anti-α-actinin 4 (green), and anti-MOMP (magenta) antibodies. Asterisks denote inclusions. (A) Confocal images of a single inclusion with actin and α-actinin 4 scaffolds. Scale bar, 30 μm. The white boxes represent a magnified section of the inclusion to show the colocalization of actin and α-actinin 4 around the inclusion (Zoom). (B, C) The 3D reconstruction of different regions of the inclusion shown in A demonstrates the colocalization of actin and α-actinin 4 around the inclusion. (D) Empty vector (Ctrl) and RhoA KO cells were infected with WT C. trachomatis L2 for 38 h (MOI, 2). Cells were fixed and labeled with anti-α-actinin 4 (red) and anti-MOMP antibodies (blue). DNA was labeled with Hoechst (gray). The white boxes represent a magnified section of the inclusion to show the recruitment of α-actinin 4 to the inclusion (Zoom). Asterisks denote inclusions. Scale bar, 25 μm. (E) The graph represents the average percentage of α-actinin 4-positive inclusions from three independent experiments ± SD. Data are normalized to Ctrl cells infected with WT C. trachomatis L2. A minimum of 100 inclusions were counted for each experiment. **, *P* < 0.01. (F) Cells were infected with WT L2 C. trachomatis L2 for 56 h (MOI, 1) and treated with ethanol (control) or 300 nM Latrunculin-B (Lat-B) for 15 min prior to fixation. Cells were then labeled with phalloidin (red), anti-α-actinin 4 (green), and anti-IncA (cyan) antibodies. Asterisks denote inclusions. Scale bar, 20 μm. The white boxes represent sections of the inclusion to show actin scaffolds and α-actinin 4 recruitment to the inclusion (Zoom).

Since α-actinins are actin-binding proteins, we used confocal microscopy coupled with three-dimensional (3D) reconstruction of the inclusion to determine whether α-actinin is also present on actin filaments surrounding the inclusion. We focused on α-actinin 4, as we can detect the endogenous protein using antibody. As shown in Fig. 2A to C, α-actinin 4 can be detected on the actin scaffolds surrounding the inclusion, where it appears to wrap around the actin filaments in a rope-like pattern.

The colocalization of α-actinin 4 with both IncA (Fig. 1A) and actin scaffolds (Fig. 2A to C) suggests that two independent pools of α-actinin 4 exist during infection. To test this possibility, we assessed α-actinin 4 recruitment in the absence of actin scaffolds by deleting RhoA, which functions downstream of InaC (11). In RhoA KO cells, C. trachomatis cannot form actin scaffolds despite the expression of InaC (8, 11). If the loss of α-actinin 4 recruitment in the InaC KO (Fig. 1C, Fig. S1B) is due to the loss of actin scaffolds, then WT-infected RhoA KO cells should phenocopy InaC KO-infected cells. Interestingly, α-actinin 4 is still recruited to ~76% of inclusions in RhoA KO cells (Fig. 2D and E). In addition, when actin scaffolds are depolymerized with Latrunculin-B (8) in wild-type cells, α-actinin 4 is still recruited to the inclusion (Fig. 2F). Together, these data indicate that (i) α-actinin 4 is present on both the inclusion membrane and actin scaffolds, (ii) the InaC-dependent recruitment of α-actinin 4 is RhoA-independent, and (iii) α-actinin 4 is still recruited to the inclusion in the absence of actin scaffolds.

### α-Actinins are not required for the formation of actin scaffolds.

**FIG 3:**
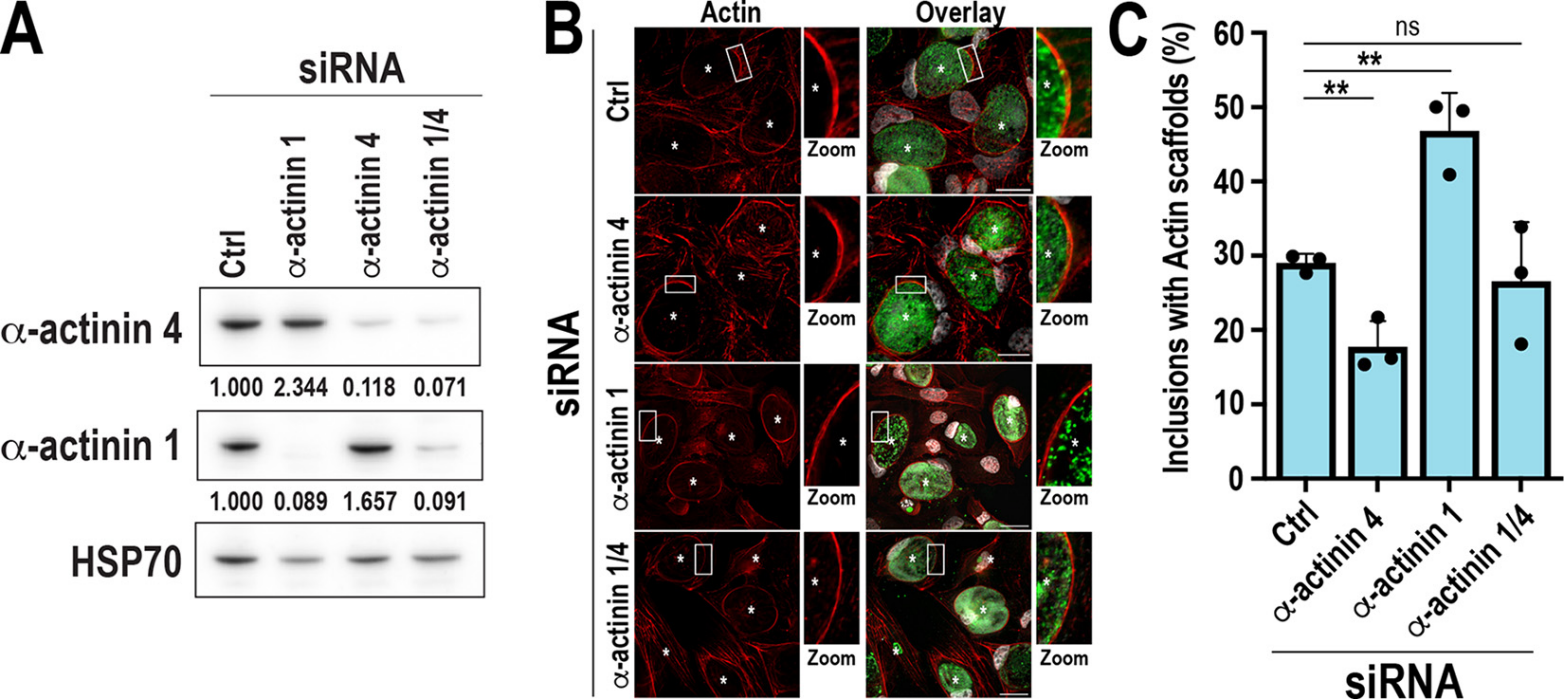
α-Actinins are not required for actin scaffold formation. (A) Cells were transfected with nontargeting (Ctrl), human ACTN1 (α-actinin 1), or human ACTN4 (α-actinin 4) siRNA for 72 h and retransfected with equivalent amounts of siRNA for 72 h. Lysates were analyzed by Western blotting to assess the degree of α-actinin knockdown. HSP70 was used as a loading control. Knockdown efficiency was determined by measuring the ratio of α-actinin:HSP70 signal normalized to Ctrl siRNA, representative of 3 independent experiments. (B) Cells were transfected with Ctrl, α-actinin 4, or α-actinin 1 siRNA for 72 h and retransfected with equivalent amounts of siRNA for 24 h prior to infection with WT C. trachomatis L2 (MOI, 1) for 46 h. Cells were fixed and labeled with phalloidin (red), anti-MOMP (green), and Hoechst (DNA, gray). Asterisks denote inclusions. Scale bar, 25 μm. The white boxes represent a magnified section of the inclusion to show actin scaffolds (Zoom). (C) The graph represents the average percentage of inclusions with actin scaffolds from three independent experiments ± SD. A minimum of 100 inclusions were counted for each experiment. **, *P < *0.01; ns, not significant.

Next, we determined whether α-actinins 1 and 4 played a role in the formation of actin scaffolds. To do so, we depleted them using small interfering RNA (siRNA) (Fig. 3A) prior to WT L2 C. trachomatis infection and measured the frequency of actin scaffolds. The loss of α-actinin 4 slightly decreased actin scaffold formation compared with the control (Fig. 3, α-actinin 4), while α-actinin 1 depletion resulted in an ~1.5-fold increase in actin scaffold formation (Fig. 3, α-actinin 1). The depletion of α-actinins did not affect α-tubulin, posttranslationally modified microtubule scaffolds, or Golgi recruitment (see Fig. S3 in the supplemental material). The simultaneous depletion of both isoforms had no impact on actin scaffold formation (Fig. 3, α-actinin 1/4), suggesting that the effects observed with single isoform depletion are likely due to the upregulation of the other isoform (Fig. 3A). In total, these data indicate that while α-actinins 1 and 4 likely contribute to actin scaffold stabilization, they are not required for their formation.

### α-Actinins promote inclusion stability during Chlamydia infection.

**FIG 4:**
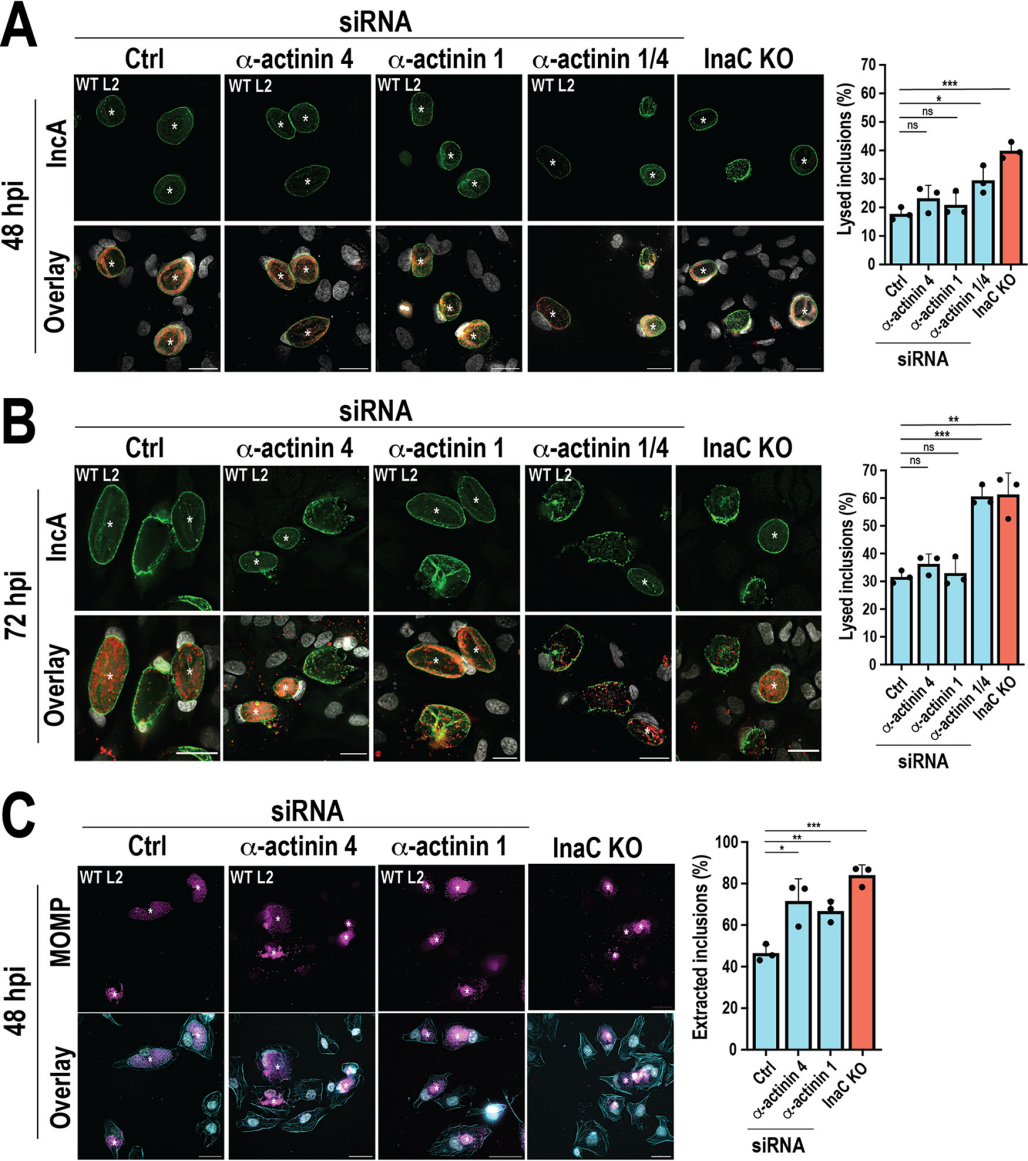
α-Actinins promote inclusion stability during Chlamydia infection. (A to C) Cells were transfected with Ctrl, α-actinin 4, or α-actinin 1 siRNA for 72 h and retransfected with equivalent amounts of siRNA 24 h prior to infection with WT or InaC KO C. trachomatis L2 (MOI, 0.5). (A, B) Cells were fixed at (A) 48 or (B) 72 hpi and labeled with anti-IncA (green) and anti-MOMP (red) antibodies. DNA was labeled with Hoechst (gray). Asterisks denote intact inclusions. Scale bar, 30 μm. The graph represents the percentage of lysed inclusions from 3 independent experiments ± SD. A minimum of 100 inclusions were counted for each experiment. (C) Prior to fixation at 48 hpi, cells were treated with 1% Triton X-100 for 5 min at 4°C. Triton was immediately removed, and the cells were fixed and labeled with phalloidin (cyan) and anti-MOMP (magenta) antibody. DNA was labeled with Hoechst (gray). Scale bar, 30 μm. The graph represents the average percentage of Triton-extracted inclusions from three independent experiments ± SD. A minimum of 100 inclusions were counted for each experiment. *, *P < *0.05; **, *P < *0.01; ***, *P < *0.001; ns, not significant.

Actin scaffolds are essential for inclusion stability (8, 11). Since α-actinins are actin-bundling/cross-linking proteins and are not required for actin scaffold formation (Fig. 3), we hypothesized that their depletion likely affects the stability of actin scaffolds and ultimately the inclusion. We tested this hypothesis using two complementary approaches (see Fig. S4 in the supplemental material). First, we assessed the susceptibility of inclusions to premature lysis. α-Actinin-depleted cells were infected with WT C. trachomatis L2 for 48 or 72 h before being stained with anti-IncA antibody to label the inclusion membrane. Inclusion membrane rupture was assessed by monitoring the continuity of the inclusion membrane (Fig. S4A and B). While individual α-actinin 1 or 4 depletion did not affect the lysis of WT C. trachomatis L2 inclusions at either time postinfection (Fig. 4A and B, α-actinin 1 or 4), simultaneous depletion of α-actinin 1 and 4 rendered inclusions more susceptible to lysis at 48 h and 72 h (Fig. 4A and B, α-actinin 1/4). As expected, InaC KO control inclusions lyse more than WT L2 inclusions (Fig. 4A and B, InaC KO) (11). Altogether, these results suggest that both α-actinin 1 and 4 contribute to maintaining inclusion membrane integrity.

The actin cytoskeleton provides morphological support to inclusions, an idea best exemplified by their resistance to detergent solubilization (8). Nonionic detergents like Triton X-100 solubilize membranes, including the inclusion membrane, while leaving stable cytoskeletal structures intact (8, 18). Because of the presence of the actin scaffolds, inclusions are largely unaffected even in the absence of the inclusion membrane. This resistance is lost upon treatment with actin-depolymerizing agents (8), highlighting the necessity of a stable actin network to maintain the inclusion compartment. If the α-actinins are important for maintaining actin scaffold stability, α-actinin depletion should render the inclusion more susceptible to detergent extraction (Fig. S4C and D).

To test this possibility, cells treated with α-actinin siRNA were infected with WT or InaC KO C. trachomatis L2 for 48 h. The infected cells were incubated with 1% Triton X-100 immediately before fixation and labeled with anti-MOMP antibody to label individual Chlamydia. We observed an ~1.5-fold increase in extraction in α-actinin 1- or α-actinin 4-depleted cells (Fig. 4C, WT L2, α-actinin 4, and α-actinin 1), demonstrating that both α-actinin 1 and 4 stabilize actin scaffolds. We could not assess α-actinin 1/4-depleted cells, as the cells themselves were highly susceptible to extraction. WT L2 inclusions are resistant to Triton X-100 extraction (Fig. 4C, WT L2 and Ctrl) (8), while InaC KO inclusions are highly susceptible to extraction (Fig. 4C, InaC KO), as these inclusions lack actin scaffolds altogether. Note that α-actinin-1 depletion results in increased susceptibility to detergent extraction despite an upregulation in actin scaffold formation (Fig. 3), indicating that these scaffolds, while more prevalent, are inherently unstable. Altogether, these data suggest that α-actinin 1 and 4 independently stabilize actin scaffolds to support inclusion integrity during infection.

## DISCUSSION

During the intracellular life cycle of Chlamydia, the inclusion grows to occupy most of the host cytoplasm. To maintain the integrity of such a large compartment, Chlamydia weaves actin filaments around the inclusion at ~32 hpi, which ultimately form a structural scaffold (8). Recently, we and others established that C. trachomatis uses the effector InaC to control the formation of these scaffolds (9–11). InaC activates the host GTPase RhoA, which is recruited on the inclusion at ~24 hpi. Activated RhoA is required for promoting actin polymerization around the inclusion as its depletion blocks this process, resulting in unstable inclusions and their premature lysis (11).

**FIG 5:**
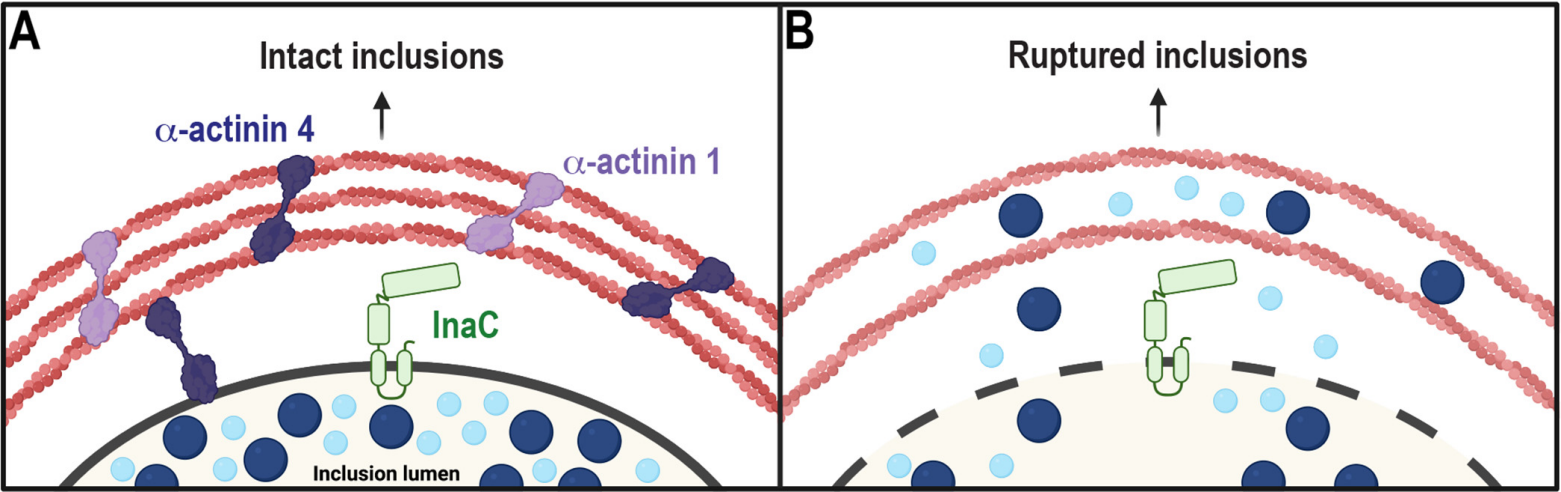
α-Actinins stabilize actin scaffolds to reinforce the inclusion membrane. (A) InaC (green) recruits α-actinin 1 (light purple) and α-actinin 4 (dark purple) where they cross-link actin scaffolds to stabilize them around the chlamydial inclusion. Consequently, these stable cytoskeletal scaffolds maintain inclusion integrity late into infection. Inclusion-associated pools of α-actinin may also play a role in regulating actin scaffold stability. (B) In the absence of the α-actinins, actin scaffolds become unstable, which leads to premature rupture of the inclusion and the release of bacteria into the cytosol. The figure was created with BioRender.com.

Here, we demonstrate that in addition to inducing the formation of actin scaffolds through RhoA, InaC recruits a new set of host effectors, the α-actinins, to further stabilize these scaffolds (Fig. 5). InaC recruits α-actinin 4 independently of RhoA, as α-actinin 4 is still present on the inclusion in RhoA KO cells (Fig. 2D and E). Interestingly, we observe α-actinin 4 on both the inclusion and the actin scaffolds. In fact, 3D reconstruction of confocal micrographs revealed that α-actinin 4 interlaces with the actin filaments that surround the inclusion (Fig. 2A to C). Ultimately, simultaneous depletion of α-actinin 1 and 4 causes premature lysis of the inclusion, and the loss of either α-actinin renders inclusions susceptible to detergent extraction, indicating that α-actinins stabilize actin scaffolds and consequently the inclusion membrane.

Since α-actinin 4 still associates with the inclusion in the absence of actin scaffolds (Fig. 2D to F), this finding suggests that the localization of α-actinin is dynamic. In this context, α-actinin would be recruited to the inclusion by InaC and shuttle between the inclusion and actin filaments as they are being generated by RhoA. Actin-bound and inclusion-associated pools of α-actinin 4 could be functionally distinct, as it would allow Chlamydia to differentially regulate α-actinin 4-dependent processes. (i) Scaffold-associated α-actinin could cross-link and bundle actin filaments to physically stabilize this structure. (ii) Inclusion-associated α-actinin could also anchor actin filaments to the inclusion membrane. Here, α-actinins could interact with actin via its actin-binding domains and with the inclusion via an unknown protein or to lipids, as α-actinins have been shown to interact with phosphoinositides (19–22). While these possibilities are not exclusive, they raise important questions as to how intracellular pathogens appropriate complex eukaryotic processes. Irrespective of whether α-actinins shuttle from the inclusion to the actin filaments, these effectors provide the inclusions with additional structural support, as the inclusions are more susceptible to detergent extraction in α-actinin-depleted cells.

The actin-independent recruitment of α-actinin suggests that Chlamydia is primed to stabilize actin scaffolds as they form. The formation of actin scaffolds is a heterogenous process, and some inclusions never form detectable actin scaffolds. Thus, each inclusion may also have unique scaffolding requirements, such as inclusion size and/or curvature, that dictate actin scaffold formation. Furthermore, the actin filaments that surround the inclusion are curved structures, which may be susceptible to physical stress, tension, and breakage. Readily available α-actinin would ensure rapid reinforcement of these scaffolds to prolong their half-life. Chlamydia precisely controls α-actinins, as these actin effectors are only recruited to the inclusion while actin scaffolds form. Altogether, our data suggest that InaC recruits α-actinins to the inclusion to provide additional stabilization to the growing chlamydial niche.

α-Actinins play signaling roles outside their canonical actin cross-linking function. Specifically, α-actinin 4 is required for Akt activation along the PI3K-Akt axis (23, 24). In this context, α-actinins associate with membranes by interacting with specific lipids (19–22, 25). This interaction allows for the recruitment of PI3K to drive the conversion of PIP2 to PIP3, effectively modifying the membrane composition. α-Actinin 1 has also been implicated in force-dependent signaling processes, which could be mediated through interactions with PIP2 (26–28). During Chlamydia infection, α-actinins could anchor actin scaffolds to the inclusion by interacting with specific lipids on the surface. Furthermore, α-actinin itself might influence the lipid composition of the inclusion by recruiting lipid-modifying proteins. These possibilities are under investigation. Ultimately, α-actinins could act beyond their cross-linking capacity as a platform for complex signaling networks at the inclusion membrane to maximize the development of the parasitic compartment during Chlamydia infection.

The role of α-actinins in bacterial pathogenesis is not well understood. Listeria monocytogenes, Salmonella enterica serovar Typhimurium, and Shigella flexneri all form actin-rich structures that regulate invasion, intracellular motility, and cell-cell spread (reviewed in references 29 and 30). α-Actinins are also recruited to many of these cytoskeletal structures (16, 17), but the precise role of α-actinins in these contexts is unknown. However, recent work has implicated α-actinin 4 in regulating invasion during Escherichia coli and Neisseria meningitidis infections (31, 32), suggesting that α-actinin 4 plays an active role in pathogenesis. Our work indicates a new role for α-actinins in stabilizing actin scaffolds during Chlamydia infection, further establishing α-actinins as regulators of bacterial pathogenesis.

Ultimately, this study also expands the role of InaC as a cytoskeleton stabilizer. We have shown previously that InaC is critical for stabilizing microtubule scaffolds and controlling their posttranslational modification. This event allows for the relocation of Golgi mini stacks around the inclusion and contributes to the growth of the inclusion. Subsequently, InaC promotes actin polymerization via RhoA. Our findings indicate yet another role for InaC in regulating the stability of actin scaffolds through the recruitment of α-actinins. Through this work, we have identified that InaC is a core stabilizer of cytoskeletal structures around the inclusion. Ultimately, this study highlights the versatility of a single chlamydial effector in the precise manipulation of multiple cytoskeletal elements.

## MATERIALS AND METHODS

### Cell culture and transfections.

HeLa cells (CCL-2; ATCC) were cultured as described previously in Dulbecco’s modified Eagle’s medium (DMEM) containing 10% fetal bovine serum (FBS), 2 mM l-glutamine, 10 μg/mL gentamicin, and nonessential amino acids (9). A2EN cells (from Alison Quayle, LSU School of Medicine) were cultured in keratinocyte serum-free medium (KSFM) containing 10% FBS, 10 μg/mL gentamicin, 2 mM l-glutamine, 3.6 mM CaCl_2_, 50 mg/L bovine pituitary extract (BPE), and 5 μg/L human recombinant epidermal growth factor (EGF). Cells were transfected with 50 nM of α-actinin 4 (ACTN4) siRNA, 10 nM of α-actinin 1 (ACTN1) siRNA, or 50 nM of nontargeting control siRNA using Dharmafect I reagent (Horizon) according to the manufacturer’s instructions. Cells were transfected once for 72 h, trypsinized, and reseeded on equivalent amounts of siRNA at 24 h before infection. Cell lysates were harvested (see “Preparation of cell lysates for Western blot analysis”) and knockdown efficiency was assessed by Western blotting (see “Western blotting”). SmartPool ON-TARGETplus nontargeting control (9), human ACTN4, and human ACTN1 (Table 1) siRNAs were purchased from Dharmacon.

**TABLE 1:** ONTARGETplus SMARTpool siRNA sequences

Gene	siRNA sequence (5′–3′)	Catalog no.
ACTN4	GACCAGAGCUGAUUGAGUA	L-011988-07
	UCGAAGUGGCUGAGAAAUA	L-011988-08
	GAGACGGGCUCAAGCUCAU	L-011988-09
	AACCAUAGCGGCCUUGUGA	L-011988-10
ACTN1	GAGACAGCCGACACAGAUA	J-011195-05
	UGACUUACGUGUCUAGCUU	J-011195-06
	GAACUGCCCGACCGGAUGA	J-011195-07
	GAAUACGGCUUUUGACGUG	J-011195-08

To assess α-actinin 1 recruitment, cells were infected with WT C. trachomatis L2 for 24 h at a multiplicity of infection (MOI) of 1 and transfected with 50 ng of pEGFP-α-actinin 1 (Addgene; no. 11908) using the Continuum transfection reagent (GeminiBio) according to the manufacturer’s instructions. Cells were fixed at 46 hpi and labeled as described in “Immunofluoresence microscopy.”

### Chlamydia strains.

Wild-type Chlamydia trachomatis serovar L2 (LGV 434/Bu) and InaC KO L2 were obtained from Ted Hackstadt (NIH, Rocky Mountain Laboratories) (9). C. trachomatis L2 was propagated and density gradient purified as described previously (33, 34). The InaC KO, InaC-FLAG, and InaC-FLAG-expressing InaC KO C. trachomatis L2 strains were generated previously (9, 11, 35).

### Antibodies.

The following primary antibodies were used: anti-α-actinin 4 (mouse, sc-393495; Santa Cruz Biotechnology [SCBT]), anti-α-actinin 1 (rabbit, ab68194; Abcam), anti-α-actinin 4 (mouse, sc-390205; SCBT), anti-α-tubulin (mouse, T5168; Sigma), anti-α-tubulin (rat, sc-53029; SCBT), anti-acetylated α-tubulin (mouse, T6793; Sigma), anti-detyrosinated α-tubulin (rabbit, 48389; Abcam), ActiStain-488 (PHDG1; Cytoskeleton), ActiStain-555 (PHDH1-A; Cytoskeleton), anti-heat shock protein 70 (HSP70) (chicken, SPC-178D; StressMarq), anti-IncA (rabbit; T. Hackstadt), and anti-MOMP (goat, 1621; ViroStat). The following secondary reagents were used: Hoechst dye (H1399), and goat and donkey anti-mouse, anti-goat, and anti-rabbit Alexa Fluor 488-, 555-, or 647-conjugated secondary antibodies (Invitrogen, Jackson ImmunoResearch). Donkey anti-chicken (IgY), anti-mouse, or anti-rabbit IgG horseradish peroxidase (HRP)-conjugated secondary antibodies were purchased from Invitrogen.

### Preparation of cell lysates for Western blot analysis.

Cells were washed with Hank’s balanced salt solution (HBSS) and lysed with ice-cold lithium dodecyl sulfate (LDS)-PAGE sample buffer (Invitrogen) containing 250 U/μL benzonase (Accelagen), 1 μg/mL pepstatin A, 5 μg/mL leupeptin, 1 mM phenylmethylsulfonyl fluoride, 10 mM sodium fluoride, and 5.4 mM sodium orthovanadate for 10 min on ice. β-Mercaptoethanol was then added to a final concentration of 0.36 M. Lysates were incubated at 95°C for 5 min and then clarified by centrifugation at 20,000 × *g* at 4°C for 10 min. Protein concentrations were determined using the Pierce 660-nm protein assay reagent containing ionic detergent compatibility reagent and read at 660 nm in a SpectraMax M2 plate reader (Molecular Devices).

### Western blotting.

Cell lysates (15 μg) were separated on 4 to 12% bis-Tris SDS-PAGE gels (Invitrogen) and transferred to polyvinylidene difluoride membranes for 1 h at 90 V and 4°C in transfer buffer (25 mM Tris, 192 mM glycine, and 10% methanol). Membranes were washed in Tris-buffered saline (TBS; 25 mM Tris base and 150 mM NaCl [pH 7.5]) and then dried at room temperature for at least 1 h. Membranes were rehydrated in methanol and washed with TBS and TBS containing 0.1% Tween 20 (TBST). Membranes were then blocked for 1 h at room temperature with blocking buffer (3% bovine serum albumin and 0.05% sodium azide in TBST). After the blocking step, membranes were incubated in primary antibody overnight at 4°C in blocking buffer. Membranes were washed with TBST before incubation with HRP-conjugated secondary antibody for 1 h at room temperature in 0.5% milk diluted in TBST. Membranes were then washed several times with TBST and TBS and revealed with SuperSignal West Dura extended duration substrate (Thermo Scientific). Membranes were imaged on a FluorChem R system (ProteinSimple), and band intensities were quantified using AlphaView software (ProteinSimple).

### Immunofluorescence microscopy.

Cells were fixed with either (i) 4% paraformaldehyde in PIPES, EGTA, magnesium, and sucrose (PEMS) buffer (80 mM PIPES, 5 mM EGTA, 2 mM MgCl_2_, and 50 mM sucrose [pH 6.8]) for 20 min at room temperature or (ii) ice-cold methanol for 10 min at room temperature. Cells were then washed in immunofluorescence-glycine (IF-G) buffer (25 mM HEPES, 150 mM NaCl, 900 nM CaCl_2_, 500 nM MgCl_2_, and 100 mM glycine [pH 7.5]) for paraformaldehyde fixation or IF buffer (25 mM HEPES, 150 mM NaCl, 900 nM CaCl_2_, and 500 nM MgCl_2_ [pH 7.5]) for methanol fixation. Permeabilization was performed with 0.2% Triton X-100 in IF buffer for 10 min, followed by washes with 0.1% Triton X-100 in IF buffer. Cells were then blocked for 1 h with either (i) goat serum (10% goat serum, 0.05% sodium azide, and 0.1% Triton X-100 in IF buffer) or (ii) donkey serum (10% donkey serum, 0.05% sodium azide, and 0.1% Triton X-100 in IF buffer) blocking buffer depending on the secondary antibodies used. After the blocking step, cells were treated with the primary antibodies diluted in the appropriate blocking buffer for 1 h at room temperature. Cells were washed with 0.1% Triton X-100 in IF buffer, subsequently incubated with Alexa Fluor-conjugated secondary antibodies and Hoechst, and diluted in the appropriate blocking buffer for 1 h at room temperature. Cells were washed with 0.1% Triton X-100 in IF buffer, followed by multiple washes with IF buffer, and mounted on coverslips with ProLong glass antifade mounting medium (Invitrogen). For the actin scaffold, α-actinin recruitment, and inclusion lysis experiments, permeabilization, blocking, and washing were performed in 0.1% saponin instead of Triton X-100.

For the Latrunculin-B experiment in Fig. 2F, cells were infected for 56 h (MOI, 1). Prior to fixation, cells were treated with ethanol (vehicle control) or 300 nM Latrunculin-B diluted in cell culture medium for 15 min at 37°C. The medium was removed, and the cells were fixed and labeled with the appropriate antibodies.

For widefield microscopy, inclusions were imaged using a Plan Apo 60×/1.4 oil immersion lens on a Nikon TiE inverted fluorescence microscope and Elements software (Nikon). For confocal microscopy, imaging was performed on a Nikon AX-R laser scanning microscope (LSCM) with an oil immersion Apo TIRF 100×/1.49 lens (160-μm working distance). Images were acquired in z-stack (widefield, 0.3 μm; confocal, 0.1 μm), and 3D reconstruction and analysis were also implemented with Elements. For all immunofluorescence experiments, representative images were deconvolved with Elements software and processed for publication using ImageJ (NIH). Actin scaffold and α-actinin positivity were determined by the presence of 75% or greater of the inclusion being surrounded by actin or α-actinin. Due to the high frequency of actin:α-actinin 4 double-positive inclusions (~85%), 100 inclusions were selected randomly for all experiments.

### Inclusion lysis analysis.

Cells transfected with nontargeting, α-actinin 4, or α-actinin 1 siRNA were infected 24 h posttransfection with WT or InaC KO C. trachomatis (MOI, 0.5) and fixed at 72 hpi. Fixed cells were labeled with anti-IncA antibody to visualize the inclusion membrane and anti-MOMP antibody to label individual bacteria. An inclusion was considered lysed when one or more substantial gaps in the incidence of IncA labeling were observed. When inclusion integrity was less clear, the presence of Chlamydia in the cytosol was used to identify a lysed inclusion (11).

### Triton X-100 inclusion extraction analysis.

Cells transfected with nontargeting, α-actinin 4, or α-actinin 1 siRNA were infected 24 h posttransfection with WT or InaC KO C. trachomatis (MOI, 0.5). At 48 hpi, culture medium was removed, and cells were treated with 1% Triton X-100 for 5 min in ice-cold HBSS containing 814 μM MgSO_4_ and 1.29 mM CaCl_2_. The buffer was then removed and immediately fixed in 4% paraformaldehyde (see “Immunofluorescence microscopy”). To determine whether an inclusion was extracted, cells were stained with phalloidin (ActiStain; Cytoskeleton) to label the cell periphery and anti-MOMP to label individual bacteria. An inclusion was considered extracted if it lacked compact morphology and if Chlamydia was detected outside the cell.

### Statistical analysis.

A two-tailed Student’s *t* test was employed when comparing the means from two independent groups. GraphPad Prism 9 was used for all statistical testing and data analysis. *P* values of <0.05 were considered statistically significant.

### Data availability.

We declare that all relevant data supporting the findings of this study are included in the manuscript and its supplemental files or are available from the corresponding author upon request.
