# Supplementary material for: Chlamydia trachomatis Subverts Alpha-Actinins To Stabilize Its Inclusion
Source: Microbiol Spectr. 2023 Jan 18;11(1):e02614-22. doi: 10.1128/spectrum.02614-22 (PMC9927245; doi:10.1128/spectrum.02614-22)

SUPPLEMENTAL FIGURE LEGENDS

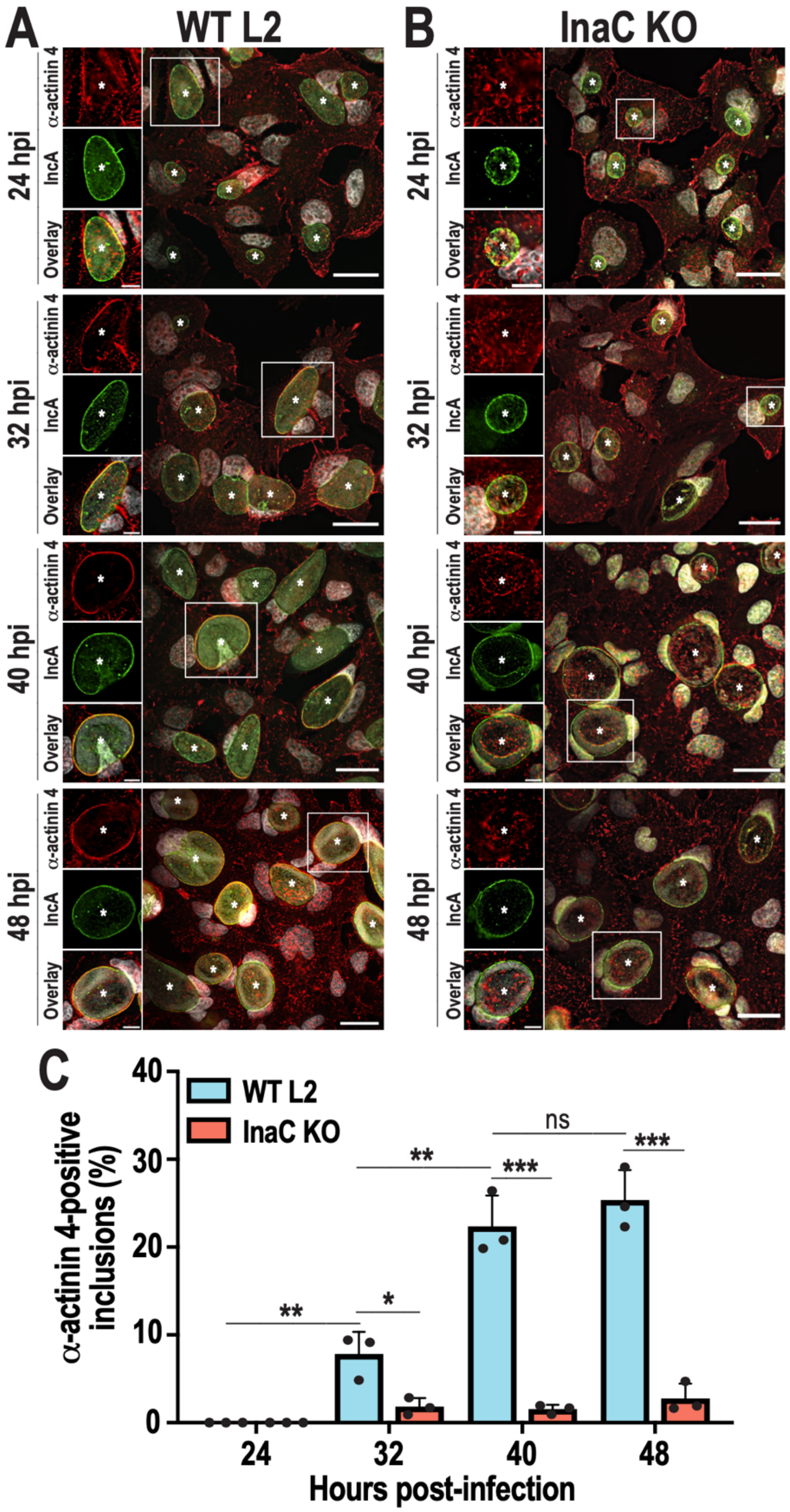

Figure S1. α-actinin 4 is recruited to the inclusion in an InaC-dependent manner late during infection.
(A-B) Cells were infected with wild-type (WT) or InaC knockout (KO) *C. trachomatis* L2 for 24-48 h (MOI 2). Cells were fixed and labeled with anti-α-actinin 4 (red) and anti-IncA (green) antibodies. DNA was labeled with Hoechst (gray). Scale bar = 30 μm. The white boxes represent a magnified image of a representative inclusion to show the recruitment of α-actinin 4. Scale bar = 10 μm. Asterisks denote inclusions. **(C)** The graph represents the average percentage of α-actinin 4-positive inclusions from three independent experiments ± the standard deviation (SD). A minimum of 100 inclusions were counted for each experiment. *, P <0.05; **, P <0.01; ***, P <0.001; ns = not significant.

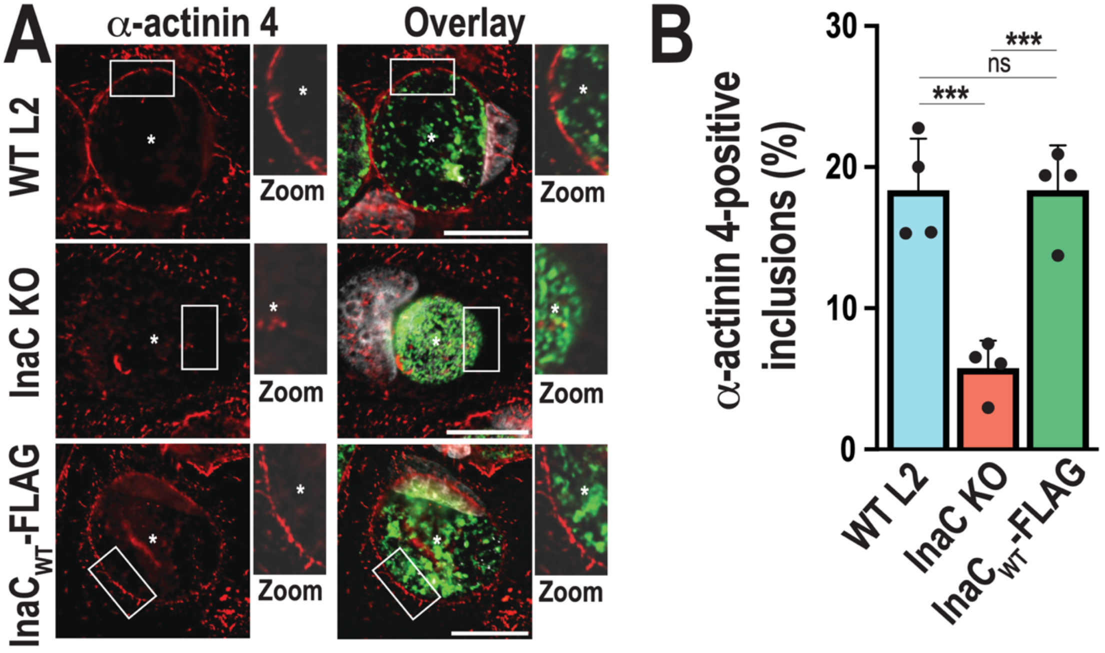

Figure S2. Complementation of InaC KO rescues the loss of α-actinin 4 around the inclusion. **(A)** Cells were infected with WT *C. trachomatis* L2, InaC KO *C. trachomatis* L2, and InaC KO *C. trachomatis* L2 complemented with InaC-FLAG (InaC$_{WT}$-FLAG) at an MOI of 1 for 46 h. InaC$_{WT}$-FLAG expression was induced 4 hpi with 5 ng/mL of anhydrotetracycline (aTc). aTc was re-added 24 hpi as it is readily degraded. Infected cells were fixed and labeled with anti-α-actinin 4 (red) and anti-MOMP (green) antibodies. DNA was labeled with Hoechst (gray). Asterisks denote inclusions. Scale bar, 30 μm. The white boxes represent a magnified section of the inclusion to show the recruitment of α-actinin 4 to the inclusion (Zoom). **(B)** The graph represents the average percentage of α-actinin 4-positive inclusions from four independent experiments ± SD. A minimum of 100 inclusions were counted for each experiment. ***, *P* <0.001; ns, not significant.

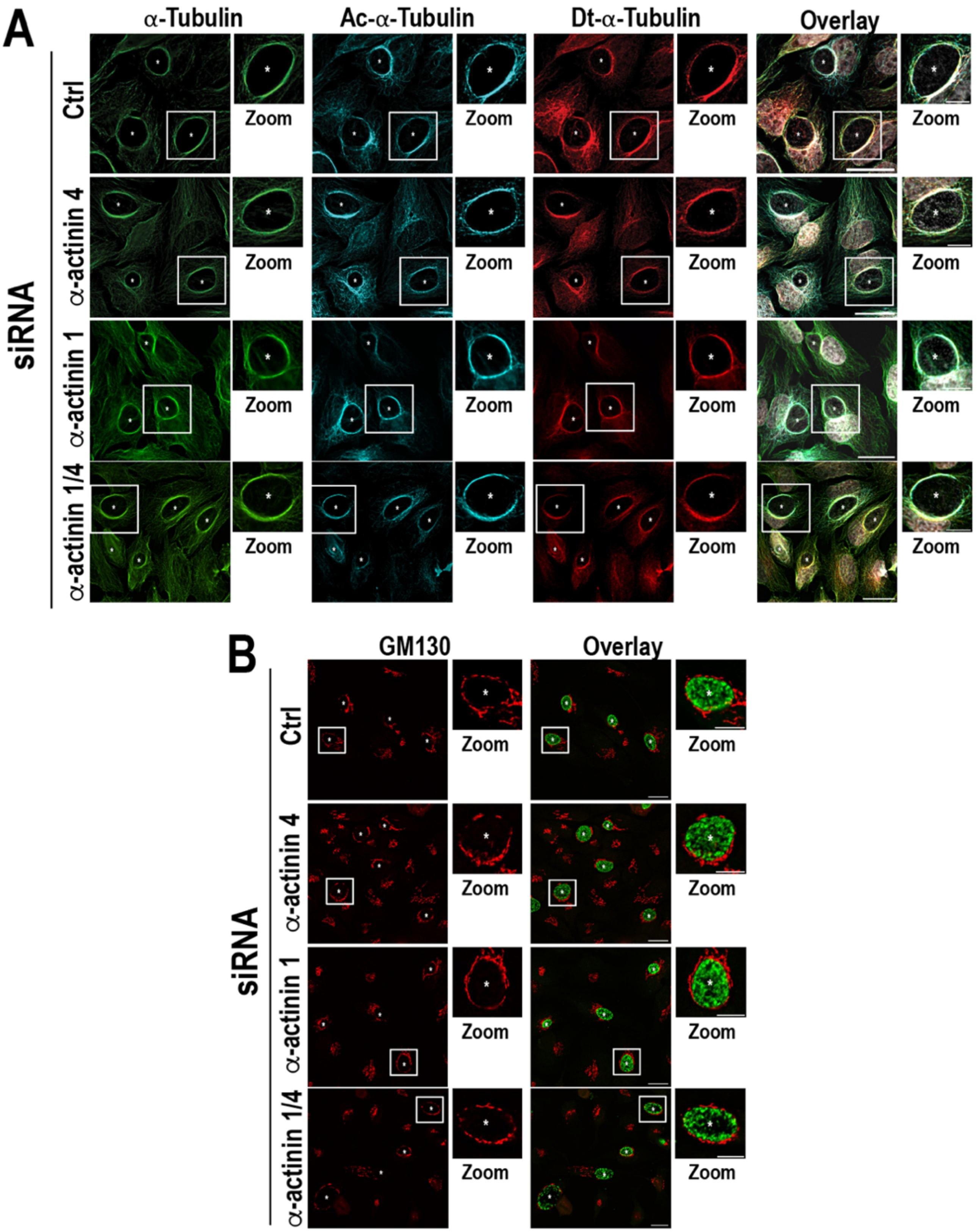
A
α-Tubulin
Ac-α-Tubulin
Dt-α-Tubulin
Overlay
siRNA
Ctrl
α-actinin 4
α-actinin 1
α-actinin 1/4
Zoom
B
GM130
Overlay
siRNA
Ctrl
α-actinin 4
α-actinin 1
α-actinin 1/4
Zoom

Figure S3. α-actinin 1 and 4 are not required for the formation of MT scaffolds or Golgi recruitment. (A-B) Cells were transfected with Ctrl, α-actinin 4, or α-actinin 1 siRNA for 72 h and re-transfected with equivalent amounts of siRNA for 24 h prior to infection with WT *C. trachomatis* L2 (MOI 2). **(A)** Cells were fixed 24 hpi and labeled with anti-α-tubulin (green), anti-acetylated-α-tubulin (cyan), and anti-detyrosinated-α-tubulin (red) antibodies. DNA was labeled with Hoechst (gray). Asterisks denote inclusions. Scale bar, 25 μm. The white boxes highlight a representative inclusion to show tubulin scaffolds (Zoom) Scale bar, 10 μm. **(B)** Cells were fixed 24 hpi and labeled with anti-α-GM130 (red) and anti-MOMP to label individual *Chlamydia* (green). Asterisks denote inclusions. Scale bar, 20 μm. The white boxes highlight a representative inclusion to show Golgi recruitment around the inclusion (Zoom). Scale bar, 10 μm.

Figure S4. Description of the inclusion extraction and lysis assays.
(A) The inclusion lysis assay tests the integrity of the inclusion membrane. In the presence of actin scaffolds, the inclusion membrane is stabilized and remains intact. In the absence of actin scaffolds, the inclusion becomes more susceptible to lysis. **(B)** Cells were infected with WT *C. trachomatis* L2 (MOI 0.5) for 48 h. Fixed cells were labeled with anti-IncA (green) antibody to visualize the inclusion membrane, and anti-MOMP (magenta) antibody to label individual *Chlamydia*. An inclusion was considered lysed when one or more substantial gaps in the incidence of IncA labeling were observed. When inclusion integrity was less clear, the presence of *Chlamydia* in the cytosol was used to identify a lysed inclusion[11]. **(C)** The Triton X-100 extraction assay takes advantage of the actin cytoskeleton's resistance to nonionic detergent extraction. Triton X-100 permeabilizes all membranes while leaving stable cytoskeletal structures intact. In the presence of stable actin scaffolds, the inclusion morphology is protected. In the presence of unstable actin scaffolds (or their absence), inclusion integrity is compromised, leaving the inclusion "extracted". **(D)** Cells were infected with WT *C. trachomatis* L2 (MOI 1) for 48 h and treated with Triton X-100 (TX-100). They were subsequently fixed and labeled with phalloidin (cyan) and anti-MOMP (magenta) antibodies to label individual *Chlamydia*. Scale bar, 20 µm. An inclusion was considered extracted if it lacked compact morphology, and/or extracellular *Chlamydia* was detected. **(A)** and **(C)** were generated with BioRender.

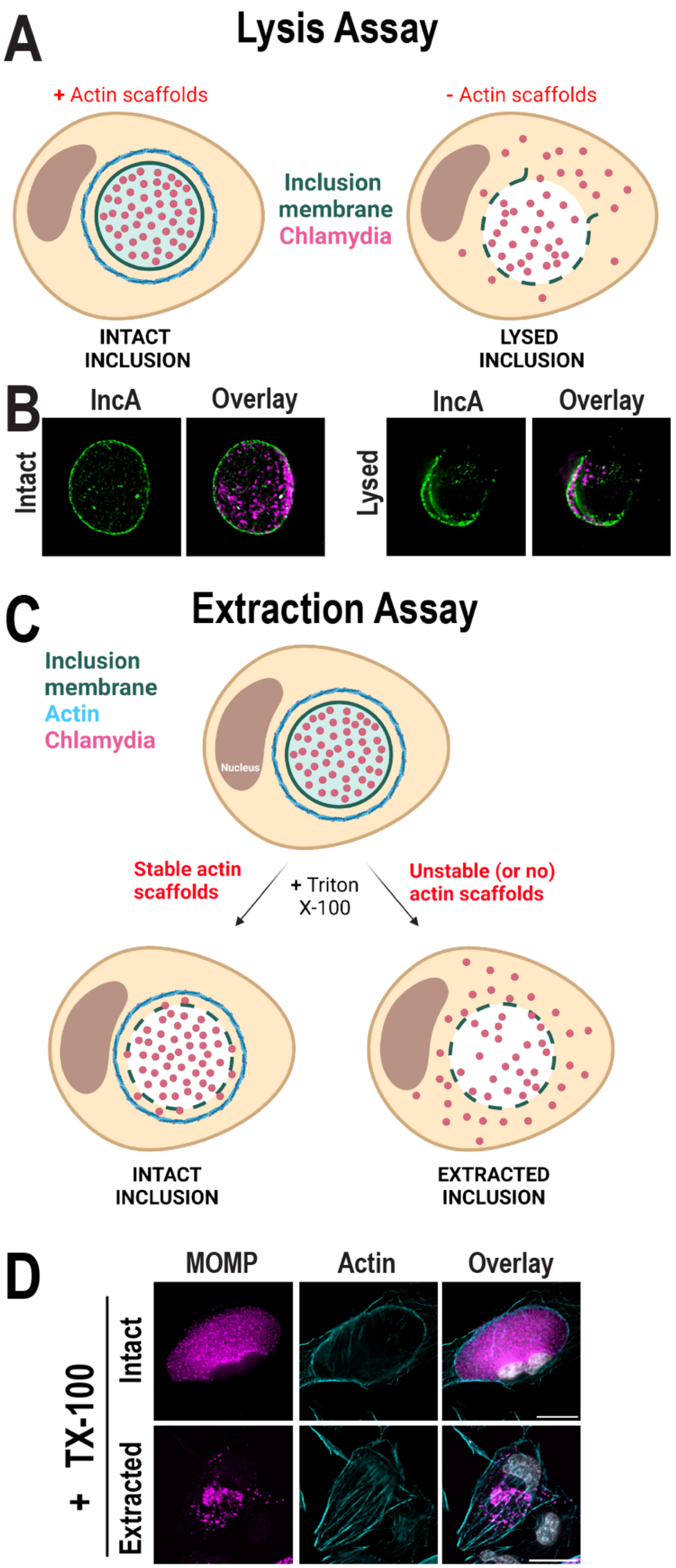

Supplement: Supplemental file 1 — Fig. S1 to S4. Download spectrum.02614-22-s0001.pdf, PDF file, 5.0 MB [file spectrum.02614-22-s0001.pdf]
